# Of mice and men: the evolution of animal welfare guidelines for cancer research

**DOI:** 10.1038/sj.bjc.6605692

**Published:** 2010-05-25

**Authors:** N Dey, P De, B R Smith, B Leyland-Jones

**Affiliations:** 1 Department of Haematology and Medical Oncology, Winship Cancer Center of Emory University, School of Medicine, University of Emory, Atlanta, GA 30322, USA; 2 VM Institute of Research, Montreal, Quebec H3A 2A5, Canada

This issue of BJC presents the revised ‘Guidelines for the welfare of animals in cancer research’ as issued by the National Cancer Research Institute (Workman *et al*, 2010). The previous efforts were published in 1988, and reissued in 1998 under the sponsorship of the United Kingdom Co-coordinating Committee on Cancer Research (UKCCCR) (Workman, 1988; Workman *et al*, 1998). These revised guidelines represent an evolution in animal use guidelines that build on and adapt to the exponential growth in scientific technique and methodology seen in cancer research over the past two decades. The use of animals for experimental investigation as an essential part of scientific enquiry has always been a significant component of medical history. Indeed, some of the earliest references for the use of animals for medical research are found in Greek writings of the second and fourth centuries BCE (Aristotle; 384–322 BCE and Erasistratus; 304–258 BCE) (Cohen and Loew, 1984). At present, animal use remains an essential part of medical science, leading to new insights into the mechanism of disease, genomic susceptibility, and new developments in diagnostics and therapy. There is also the widespread recognition of the ethical and moral imperative to conduct this research work, with the highest standards seeking to balance the desire for scientific clarity with a major emphasis on the concern for animal welfare. The revised ‘Guidelines for the Welfare and Use of Animals in Cancer Research’ published in this issue is a direct outgrowth of this recognition.

Historically, cancer has earned a reputation as a deadly disease accompanied by pain and suffering in humans. Although medical science has made great strides in the prevention and treatment of cancer, it still remains that one in three individuals in the developed world will develop some form of cancer in their lifetime. In the pursuit to alleviate this condition in our species, oncological research makes use of the full panoply of technological tools available to elucidate the signs and symptoms, causes, pathophysiology, diagnostic insights, treatment, and epidemiology of cancer. Yet, despite the substantive advancements in the use of *in vitro* models, we do not have a fully effective alternative to the use of *in vivo* study for the examination of the fundamental processes of this disease in living organisms.

Virtually every important medical achievement in the twentieth century has relied on the use of animals in some way (The Royal Society, 2004). Oncology research has a similar history, with the discovery of some 24 significant biomedical advances in the past 30 years that would likely not have occurred without animal experimentation (Frankie, 2005). However, it is abundantly clear that this research effort can be conducted effectively in such a manner as to reduce the adverse effects on animals to a minimum. The revised ‘Guidelines for the Welfare and Use of Animals in Cancer Research’ provide the framework for the maintenance of the highest standards of scientific enquiry in conjunction with concern for the welfare of the subjects used in this endeavour.

The fundamental problem of animal welfare in cancer research lays in its inherent dichotomy. Animal models are developed to test the efficacy of potential life-saving agents against a painful and fatal disease, cancer. The inherent discordance from an animal welfare prospective is that animals must be exposed to this disease in some manner to provide a model of the mechanisms of the progression of cancer in humans. Yet, without laboratory animals (especially athymic mice; ∼95% of all animal studies are conducted in mice), cancer researchers would lose a fundamental method for obtaining data required to make appropriate decisions about potential new therapies that would have enormous impact in human populations.

The key principles that have served to provide a framework for subsequent legislative and scientific efforts in guiding animal use in laboratory research were first published by Russell and Burch (1959). Their postulation of the ‘Principles of Humane Experimental Techniques’ argued that the adoption of the ‘3Rs’ (Replace, Reduce, or Refine) should be an integral component of scientific planning and execution in all studies involving *in vivo* animal use. In the opinion of Goldberg and Locke (2004), ‘Humane science based on the principles of the 3Rs can mean better scientific results’. The standard rodent models for carcinoma used for the past 30 years to optimise therapy in humans consist of either s.c. xenograft or orthotropic implantation of human tumour cells into athymic mice. These models have several characteristics that make them good testing systems, including defined and reproducible location of tumour formation, rate of tumour growth, and progression of disease (Finkelstein *et al*, 1994). Following one of the tenets of the 3Rs (Russell and Burch, 1959), refinement, several laboratories have developed genetically and histologically accurate models of carcinoma by gain-of-function (transgenic) approaches and targeted deletion (including tissue-specific) strategies. Transgenic mice express the gene of interest in all cells (Aguzzi *et al*, 1995), whereas knockout mice lose expression of the targeted gene in all cells that would normally express it (Macleod and Jacks, 1999). These are gene-manipulated ‘extra-evolutionary species’ of mice bred for medical/scientific purposes.

An example of the influence of another tenet of the 3Rs, replacement, is the recent development of the ‘Biobank’ (BC, Canada) as a repository for the transfer of bio-specimens (tissues, blood, body fluids) and related data that can serve as an interface between researchers seeking bio-specimens and clinical data related to different aspects of disease progression (Watson *et al*, 2009). The establishment of similar biobanks of human biopsy tissue coupled with clinical data is accelerating throughout the world and holds the promise of yielding translational discoveries in areas such as novel molecular and gene targets and candidate protein biomarkers of disease progression and outcome.

The last of the 3Rs, reduction, is reflected in the development of scientific methodologies that minimise the number of experimental animals required to provide sufficient statistical power and seek to maximise the data obtained from each individual life. In many European countries including the United Kingdom, such efforts have witnessed a decline in animal use by >20% between 1991 and 2002 (Frank, 2008). One of the major alternatives to *in vivo* modelling is the development of ‘*in silico*’ computer simulation. It is encouraging and worth noting, that *in silico* modelling has shown some promise as another potential tool in understanding the underlying mechanisms of tumour biology and the development of predictions of tumour response to different drugs (such as cisplatin and doxorubicin). Pharmacokinetics and pharmacodynamics of early-stage compounds are increasingly being modelled using computer simulations because of its ability to integrate phenomena such as vascular extravasation of oxygen and drugs, interstitial diffusion, and cellular response to local concentrations in spatially multidimensional settings (Sanga *et al*, 2006; Sinek *et al*, 2009). However, despite these *in silico* advances, they are not yet able to act as complete alternatives to *in vivo* animal experimentation due to the fact that the simulations cannot examine the entire ‘chemical space’ of living complex organisms (Lipinski and Hopkins, 2004). At present, sophisticated computer simulations, while improving at an exponential rate, are unable to model the myriad of interactions between molecules, cells, tissues, organs, and the environment, leaving animal research a necessary component of oncology research (Committee to Update Science, 2004). Having said that, ‘*in silico* computer simulation’ can reduce animal usage for testing of new chemical entities with large combinatorial libraries As we are still far from an ‘*in silico* mouse’, the choice for testing anticancer drugs remains with *in vitro*, *ex vivo*, *in silico* computer simulation and meticulously designed *in vivo* models.

The incidence of cancer is projected to increase worldwide (Boyle, 1997). Thus, there is immense social and economic pressure to develop new and more efficacious anticancer therapies. Cancer research is a global enterprise, with medical and scientific enquiries occurring in a wide range of jurisdictions around the world, each with their own regulatory mandates and concerns. As scientific progress will continue at an accelerated pace in cancer research, it would be expected that consensual and enforceable ethical guidelines would continue to evolve for the betterment of this research with due respect to laboratory animal use. Given that animal experimentation will, for the time being, remain indispensable in cancer/biomedical research, then the application of principles towards the minimal use of experimental animals following the 3Rs (Russell and Burch, 1959) will continue to serve as an accepted framework to ensure that the welfare of animals in cancer research is protected. The ‘Guidelines for the Welfare of Animals in Cancer Research’ (Workman *et al*, 2010) published in this issue is a continuation of this evolution. Taking into account the continuous advancement of medical technologies and new methodologies, the revised Guidelines provide current and future investigators, governmental regulatory agencies, and pharmaceutical industry with the necessary information to ensure that their research activities follow the highest possible standards of animal welfare that is consistent with not only ethical and moral imperatives but also good scientific practice.
